# Effect of oxygenation modalities among patients with postoperative respiratory failure: a pairwise and network meta-analysis of randomized controlled trials

**DOI:** 10.1186/s40560-020-00468-x

**Published:** 2020-07-17

**Authors:** Yazan Zayed, Babikir Kheiri, Mahmoud Barbarawi, Laith Rashdan, Inderdeep Gakhal, Esra’a Ismail, Josiane Kerbage, Fatima Rizk, Saadia Shafi, Areeg Bala, Shima Sidahmed, Ghassan Bachuwa, Elfateh Seedahmed

**Affiliations:** 1 Department of Internal Medicine, Hurley Medical Center/Michigan State University, One Hurley Plaza, Suite 212, Flint, MI 48503 USA; 2 Knight Cardiovascular Institute, Oregon Health & Science University, Portland, Oregon USA; 3 College of Human Medicine, Michigan State University, East Lansing, MI USA; 4 Department of Anesthesia, Lebanese University, Beirut, Lebanon; 5 College of Osteopathic Medicine, Michigan State University, East Lansing, MI USA; 6 Department of Pulmonary and Critical Care, Hurley Medical Center/Michigan State University, Flint, MI USA

**Keywords:** Postoperative respiratory failure, High-flow nasal cannula, Non-invasive ventilation, Standard oxygen, Meta-analysis

## Abstract

**Background:**

Postoperative respiratory failure is associated with increased perioperative complications. Our aim is to compare outcomes between non-invasive ventilation (NIV), high-flow nasal cannula (HFNC), and standard oxygen in patients at high-risk for or with established postoperative respiratory failure.

**Methods:**

Electronic databases including PubMed, Embase, and the Cochrane Library were reviewed from inception to September 2019. We included only randomized controlled trials (RCTs) that compared NIV, HFNC, and standard oxygen in patients at high risk for or with established postoperative respiratory failure. We performed a Bayesian network meta-analysis to calculate the odds ratio (OR) and Bayesian 95% credible intervals (CrIs).

**Results:**

Nine RCTs representing 1865 patients were included (the mean age was 61.6 ± 10.2 and 64.4% were males). In comparison with standard oxygen, NIV was associated with a significant reduction in intubation rate (OR 0.23; 95% Cr.I. 0.10–0.46), mortality (OR 0.45; 95% Cr.I. 0.27–0.71), and intensive care unit (ICU)-acquired infections (OR 0.43, 95% Cr.I. 0.25–0.70). Compared to standard oxygen, HFNC was associated with a significant reduction in intubation rate (OR 0.28, 95% Cr.I. 0.08–0.76) and ICU-acquired infections (OR 0.41; 95% Cr.I. 0.20–0.80), but not mortality (OR 0.58; 95% Cr.I. 0.26–1.22). There were no significant differences between HFNC and NIV regarding different outcomes. In a subgroup analysis, we observed a mortality benefit with NIV over standard oxygen in patients undergoing cardiothoracic surgeries but not in abdominal surgeries. Furthermore, in comparison with standard oxygen, NIV and HFNC were associated with lower intubation rates following cardiothoracic surgeries while only NIV reduced the intubation rates following abdominal surgeries.

**Conclusions:**

Among patients with post-operative respiratory failure, HFNC and NIV were associated with significantly reduced rates of intubation and ICU-acquired infections compared with standard oxygen. Moreover, NIV was associated with reduced mortality in comparison with standard oxygen.

## Introduction

Postoperative respiratory failure is associated with increased perioperative complications such as reintubation, invasive mechanical ventilation, and healthcare-associated infections, which can lead to increases in mortality, intensive care unit (ICU) and hospital length of stay, delays in hospital discharges, and higher healthcare resource utilization [1–4].

Several post-operative pulmonary complications may result in post-operative hypoxemic respiratory failure, including pneumonia, atelectasis, bronchospasm, pneumothorax, and pleural effusion. The incidence of these complications is variable and ranges between 5 and 40% according to the type of surgery, as well as other risk factors including anesthetic technique, duration of surgery, and severity of illness [5–9]. Cardiac surgery has the highest rate of post-operative respiratory complications (up to 40%), followed by thoracic surgery (30%), while abdominal and vascular surgeries have a low incidence of post-operative pulmonary complications (6–7%) [5–7].

In nonsurgical patients, oxygenation modalities for hypoxemic respiratory failure are varied. Non-invasive ventilation (NIV) has shown promising results for reducing intubation rates among patients with cardiogenic pulmonary edema and chronic obstructive pulmonary disease exacerbations [10–12]. High-flow nasal cannula (HFNC) is a new oxygenation strategy that delivers oxygen at high concentrations and a high flow rate and has been increasingly utilized due to its ease of application, tolerance, and potential clinical benefits [13, 14]. Nevertheless, the current European Respiratory Society/American Thoracic Society (ERS/ATS) guidelines have conditional recommendations regarding the use of NIV in postoperative respiratory failure [15]. In addition, few randomized controlled trials (RCTs) have been conducted to evaluate the efficacy of HFNC vs NIV in postoperative patients. Therefore, we conducted a meta-analysis to compare the efficacy and safety of HFNC, NIV, and standard oxygen therapy in the treatment of patients who developed or were considered high risk for post-operative respiratory failure.

## Methodology

### Study design and study selection

Our study is a meta-analysis and systematic review performed according to the Preferred Reporting Items for Systematic Reviews and Meta-Analyses Protocols (PRISMA-P) 2015 Statement [16]. Two reviewers (M.B., I.G) independently and separately performed a literature search utilizing electronic databases including PubMed, Cochrane Library, and Embase from inception to September 2019 without language restrictions. Articles were first screened by titles and abstracts before exclusion. Full texts of eligible articles were reviewed for final inclusion or exclusion. Mesh terms used were as follows: “postoperative respiratory failure”, “respiratory failure”, “postoperative”, “hypoxemic”, “hypoxic”, “non-invasive ventilation”, “NIV”, “high-flow nasal cannula”, “HFNC”, “high-flow nasal therapy”, “HFNT”, “high-flow nasal oxygen”, “HFNO”, “oxygen”, “facemask”, and “ventilation”. References of relevant articles were also reviewed for possible inclusion. A third reviewer (YZ) resolved any discrepancies.

### Inclusion criteria and study selection

Only RCTs were eligible for inclusion in our analysis. We included studies that compared different oxygenation strategies in patients who developed or were deemed at high risk for developing post-operative hypoxemic respiratory failure. Patients at risk were defined to have intermediate to high risk for development of post-operative pulmonary complications according to either Assess Respiratory Risk in Surgical Patients in Catalonia (ARISCAT) score of ≥ 26 points [4]. Patients who failed spontaneous breathing trial and those who passed spontaneous breathing trials but had risk factors for failed extubation such as cardiac dysfunction, obesity (BMI > 30), or failure of previous extubation were also considered high risk. Post-operative hypoxemic respiratory failure was defined as the development of tachypnea with a respiratory rate of ≥ 25 respirations per minute, intense work of breathing with the use of accessory muscles, hypoxemia (oxygen saturation ≤ 92% or partial arterial oxygen pressure to fraction of inspired oxygen ≤ 300) in the immediate post-operative period or within 7 days post-operatively. We excluded studies that investigated prophylactic use of NIV and HFNC as a routine therapy in the post-operative period.

Data were extracted into a predesigned table independently and separately by two reviewers (L.R and S.S.). Any discrepancies were solved by consensus with a third reviewer (Y.Z.).

### Quality assessment

Cochrane Collaboration’s tool for assessing risk of bias in randomized controlled trials was used for quality assessment for the included RCTs [17]. Each of the included RCTs was assessed for random sequence generation, allocation concealment, blindness of participants and health-care personnel, blindness of outcome assessment, incomplete outcome data, selective reporting, and other biases if any were present.

### Outcomes

Our main outcome was the intubation rate following surgery. Secondary outcomes included mortality at the longest follow-up period provided by each study and ICU-acquired infections.

### Statistical analysis

An informative prior Bayesian framework for the network meta-analysis was performed using the Markov Chain Monte Carlo simulation to derive the posterior distribution of the parameter estimates. We used a beta distribution of (0, 2) for binominal likelihood. We used the Brooks-Gelman-Rubin method to assess for convergence. A consistency model which contains treatment as a fixed effect and trial as a random effect was used. Results were reported as odds ratios (ORs) and Bayesian 95% credible intervals (Cr.Is). Inconsistency was assessed using the deviance residuals and deviance information criteria statistics. Sensitivity analysis was performed by including only trials that included patients who had developed respiratory failure. Furthermore, subgroup analysis according to the type of surgery (cardiothoracic or abdominal) was performed. In addition, to show the validity of our results, we performed a direct pairwise meta-analysis for comparisons that have three or more studies comparing directly the two interventions.

In an exploratory analysis, we performed a meta-regression analysis to explain any significant heterogeneity (> 25%) for NIV vs standard oxygen therapy direct meta-analysis. Moderators included study-level covariates: age, gender, body mass index, Simplified Acute Physiology Score (SAPS) II, respiratory rate, PaO2/FiO2 ratio, and partial arterial pressure of carbon dioxide (PaCO2). All data were analyzed using RevMan v5.3 Windows, Comprehensive Meta-Analysis software v3, NetMetaXL v1.6.1, and WinBUGS v1.4.3.

## Results

### Summary of the included studies

**Fig. 1:**
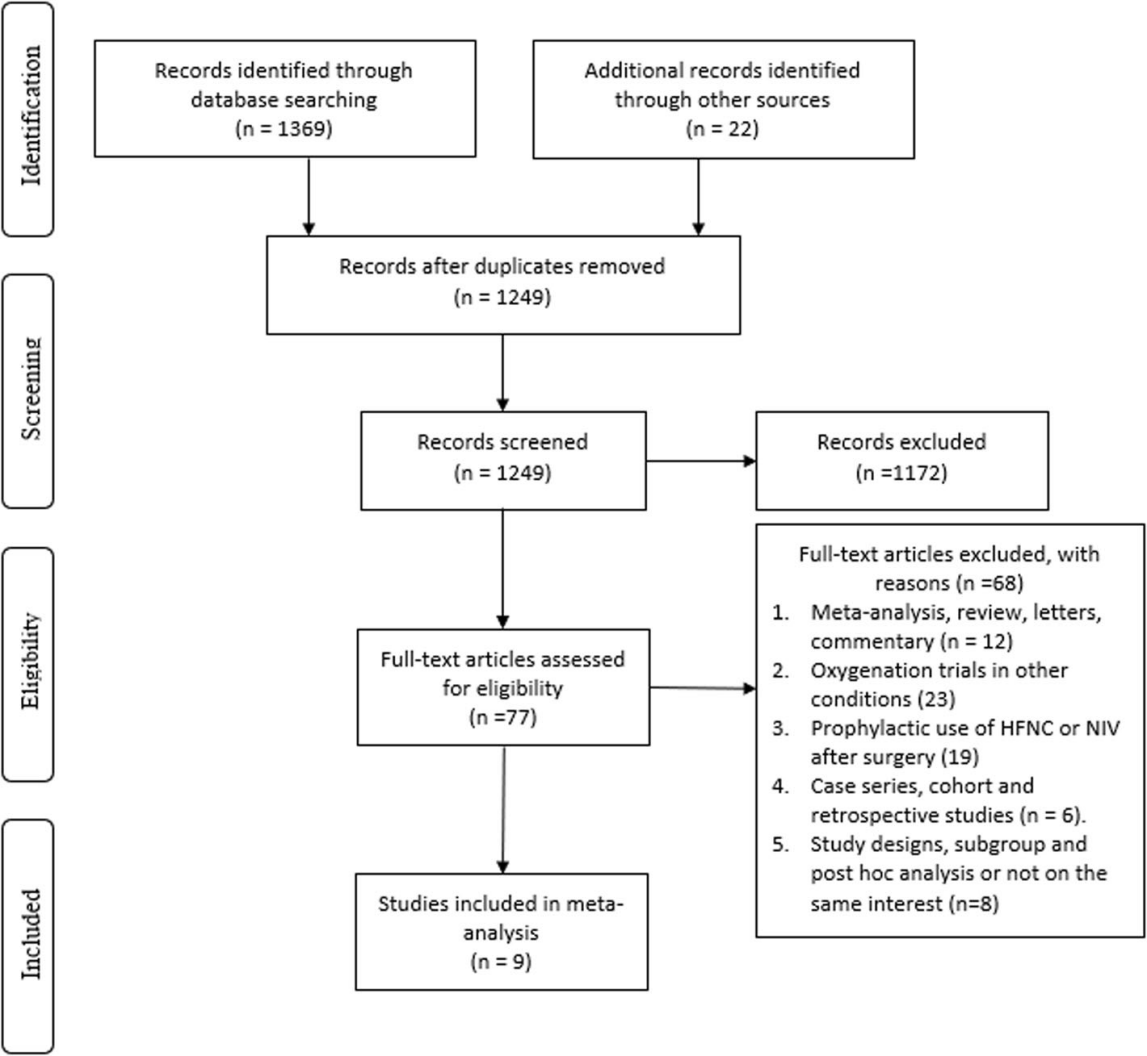
Flow chart of literature search and study selection

After review of 1369 articles, 9 studies were included in the final analysis representing 1865 patients [18–26]. Figure 1 illustrates the search process. The mean age was 61.6 ± 10.2, and 64.4% were males. Four RCTs included patients undergoing cardiac and/or lung surgeries [19, 20, 24, 26], 3 RCTs involved patients undergoing abdominal surgeries [21–23], and 2 RCTs included patients following organ transplantation [18, 25]. Two trials included patients considered at high risk of post-operative pulmonary complications and respiratory failure [23, 26] and one trial included patients at risk for respiratory failure or patients with established respiratory failure [20] while six trials included patients who developed respiratory failure in the immediate post-operative period or up to 7 days postoperatively [18, 19, 21, 22, 24, 25]. Two trials compared HFNC vs NIV [18, 20], five trials compared NIV vs standard oxygen therapy [19, 21, 22, 24, 25], and 2 trials compared HFNC vs standard oxygen therapy [23, 26]. Table 1 explains the characteristics of the included trials, and Supplementary Figure 1 illustrates the network geometry. NIV was the most commonly used treatment (41.2% of patients), HFNC was used in 31.6% of cases, and 27.2% of patients were treated with standard oxygen therapy. Table 2 explains the baseline and demographic characteristics of included patients.

**Table 1:** Characteristics of the included studies

Study (author, year)	Study groups	Study design	Inclusion criteria	Settings of experimental group and control group intervention	Follow-up period
Yu, 2017	HFNC 56, SO 54	Multicenter, prospective, randomize, interventional trial	Patients who underwent thoracoscopic lobectomy because of lung tumor and were at intermediate to high risk for PPC as determined by an ARISCAT score ≥ 26. Patients were immunocompetent, not pregnant, between 18 and 80 years old	HFNC: received at a flow rate if 35 to 60 L/min and FiO2 was titrated from 45 to 100% to maintain a SpO2 of 95% or more SO: received oxygen via nasal prongs or facemasks with FiO2 titrated between 45 and 100% to maintain SpO2 of 95% or more	72 h following extubation
Futier, 2016	HFNC 108, SO 112	Multicenter, randomized controlled trial	Adult patients scheduled for planned or unplanned abdominal, or abdominal and thoracic surgery with and anticipated duration of 2 h or more and an ARISCAT score ≥ 26	HFNC: flow rate of 50 to 60 L/min to maintain an SpO2 of 95% or more SO: O2 delivered continuously using nasal prongs or facemasks to maintain an SpO2 of 95% or more	7 days post-op
Gupta, 2016	HFNC 10, NIV 10	Pilot study, single-center, randomized controlled trial	Postoperative hypoxemia in post-liver transplant patients	HFNC: initiated at a flow rate of 60 L/min and titrated according to ABG NIV: set EPAP of 5 cm and IPAP at 10 cm and titrated according to ABG	48 h post-op.
Jaber, 2016	SO 145, NIV 148	Multicenter, randomized, parallel-group clinical trial	Patients older than 19 who had undergone laparoscopic or non-laparoscopic elective or nonelective abdominal surgery under general anesthesia that were diagnosed with ARF within 7 days of surgical procedure defined as persistence of more than 30 min of hypoxemia	SO: supplemental O2 at a rate of up to 15 L/min to maintain SpO2 of at least 94% NIV: facemask connected to an ICU or NIV dedicated ventilator titrating PEEP and FiO2 to maintain an SpO2 of at least 94%	90 days post-op.
Stephan, 2015	HFNC 414, NIV 416	Multicenter, randomized, noninferiority trial	Patients who had undergone cardiothoracic surgery who developed ARF (failure of SBT or successful SBT but failed extubation) or were deemed at risk for respiratory failure post-extubation due to preexisting risk factors	HFNC: initial rate of 50 L/min with initial FiO2 50% adjusted to maintain SpO2 92% or more BiPAP: full facemask connected to ventilator with adjustments made to PEEP and FiO2 to maintain SpO2 of 92% or more	3 days
Zhu, 2013	NIV 48, SO 47	Single-center, prospective, randomized control study	Patients who after cardiac surgery developed ARF after initial extubation who were hemodynamically stable with no evidence of bleeding	NPPV: BiPAP via facemask. FiO2 adjusted to maintain SpO2 of around 92% SO: standard medical care and oxygen therapy as needed	Length of hospital stay
Squadrone, 2005	NIV 105, SO 104	Multicenter, randomized, controlled, unblinded study	Post-op elective abdominal surgery under GA if surgery required laparotomy and time of viscera exposure longer than 90 min. Patients were extubated after surgery, and if they developed a PaO2/FiO2 of 300 less, they were included in study.	CPAP: treated with FiO2 of 0.5 plus CPAP of 7.5. After 6 h, patients underwent 1-h screening test breathing O2 through a venture mask at an FiO2 of 0.3. Patients returned to assigned treatment if PaO2/FiO2 ratio was 300 or less, and treatment was interrupted if the ratio was higher than 300 SO: 8 to 10 L/min oxygen.	Length of hospital stay
Auriant, 2001	NIV 24, SO 24	Prospective, randomized controlled trial	Patients with AHRI following lung resection if they met at least three of the following criteria: dyspnea at rest, active contraction of accessory respiratory muscles, PaO2/FiO2 less than 200, chest radiographic abnormalities	NPPV: cushion bridge nasal mask with BiPAP. PS was increased to achieve exhaled TV of 8–10 mL/kg and RR of less than 25 breaths/min. FiO2 was adjusted to obtain SpO2 above 90% SO: O2 supplementation to achieve SaO2 above 90%	120 days
Antonelli, 2000	NIV 20, SO 20	Single center, prospective, randomized study	Recipients of solid organ transplants with acute hypoxemic respiratory failure. Criteria included acute respiratory distress, respiratory rate greater than 35/min, ratio of PaO2/FiO2 of less than 200, active contraction of accessory muscles or paradoxical abdominal motion	NIV: ventilator connected to full-face mask with titration of PS to obtain exhaled TV of 8 to 10 mL/kg, RR less than 25/min. PEEP increased gradually and up to 10 cm H_2_O until FiO2 requirement was 0.6 or less. Settings were adjusted based on continuous oximetry and measurements of ABG. Standard oxygen: Venturi mask started with FiO2 of 40% and titrated to achieve a level of SpO2 90%	NA

*HFNC* high-flow nasal cannula, *SO* standard oxygen, *NIV* non-invasive ventilation, *PPC* postoperative pulmonary complications, *ARISCAT* assess respiratory risk in surgical patients in Catalonia, *FiO2* fraction of inspired oxygen, *SpO2* peripheral capillary oxygen saturation, *L* liters, *min* minute, *ABG* arterial blood gas, *EPAP* expiratory positive airway pressure, *IPAP* inspiratory positive airway pressure, *ARF* acute respiratory failure, *ICU* intensive care unit, *PEEP* positive end-expiratory pressure, *BiPAP* bilevel positive airway pressure, *SBT* spontaneous breathing trail, *CPAP* continuous positive airway pressure, *PaO2* partial pressure of oxygen, *AHRI* acute hypoxemic respiratory insufficiency, *mm* millimeter, *Hg* mercury, *TV* tidal volume, *PS* pressure support

**Table 2:** Baseline demographic and clinical characteristics of included patients

Study name	Study groups	Total number	Age	Male (%)	BMI	SAPS II score	Respiratory rate	PaO2/FiO2 ratio	PaCO2
**Yu 2017**	HFNC	56	56.31 ± 7.03	54	26.32 ± 4.73	NA	18.43 ± 3.45	350 ± 33.87	41.73 ± 6.33
	SO	54	55.82 ± 7.92	52	25.19 ± 5.02	NA	17.98 ± 3.87	341 ± 40.65	43.52 ± 4.93
**Jaber 2016**	NIV	148	62.5 ± 14.5	78.4	27.2 ± 5.9	33.6 ± 12.8	28.2 + 7.7	201 ± 69	39 ± 7
	SO	145	64.4 ± 13.1	74.5	27.1 ± 6.2	33.4 ± 11.7	28.8 + 7.3	188 ± 71	37 ± 7
**Gupta 2016**	HFNC	10	NA	NA	NA	NA	NA	NA	NA
	NIV	10	NA	NA	NA	NA	NA	NA	NA
**Futier 2016**	HFNC	108	62 ± 12	56	25 ± 4	NA	NA	NA	NA
	SO	112	61 ± 13	57	25 ± 4	NA	NA	NA	NA
**Stephan 2015**	NIV	416	63.9 (62.6–65.2)	67	28.2 (27.6–28.7)	28.8 (27.7–30.0)	23.2 (22.6–24.0)	203 (195–212)	39.1 (38.4–39.8)
	HFNC	414	63.8 (62.5–65.2)	66	28.3 (27.8–28.8)	29.0 (27.8–30.1)	22.8 (22.1–23.5)	196 (187–204)	38.7 (38.1–39.4)
**Zhu 2013**	NIV	48	62 ± 10.3	66	25.3 ± 4.6	NA	28.3 ± 8.6	NA	38.9 ± 12.2
	SO	47	61 ± 12.2	57	24.4 ± 3.5	NA	25.4 ± 6.7	NA	38.3 ± 11.3
**Squadrone 2005**	NIV	105	66 ± 9	68	26.5 ± 4.7	27 ± 7	NA	247 ± 33	39 ± 7
	SO	104	65 ± 10	62	26.3 ± 4.5	28 ± 8		255 ± 31	39 ± 5
**Aurian 2001**	NIV	24	58.9 ± 10	NA	NA	16.9 ± 5.4	26.25 ± 13.2	124 ± 50.2	63.9 ± 20.5
	SO	24	63 ± 9	NA	NA	16.8 ± 4.4	29.5 ± 6.9	111 ± 54.3	43.4 ± 9.3
**Antonelle 2000**	NIV	20	45 ± 19	65	NA	NA	38 ± 3	NA	42 ± 10
	SO	20	44 ± 10	60	NA	NA	37 ± 1	NA	38 ± 10

Data are provided percent (%), mean ± SD, or median (interquartile range)

*HFNC* high-flow nasal cannula, *SO* standard oxygen, *NIV* non-invasive ventilation, *BMI* body mass index, *SAPS* simplified acute physiology score, *PaO2/FiO2* partial pressure of arterial oxygen to fraction of inspired oxygen ratio, *PaCO2* partial pressure of arterial carbon dioxide, *NA* not available

Included studies were noted to have inevitable performance bias as blinding of participants and personnel was difficult given the nature of the intervention. Detailed quality assessment was not performed for one study as we only found the abstract with no full article explaining the methods. Supplementary Figure 2 shows the risk of bias in each included RCT based on the authors’ judgment.

### Outcomes

#### Rate of intubation

**Fig. 2:**
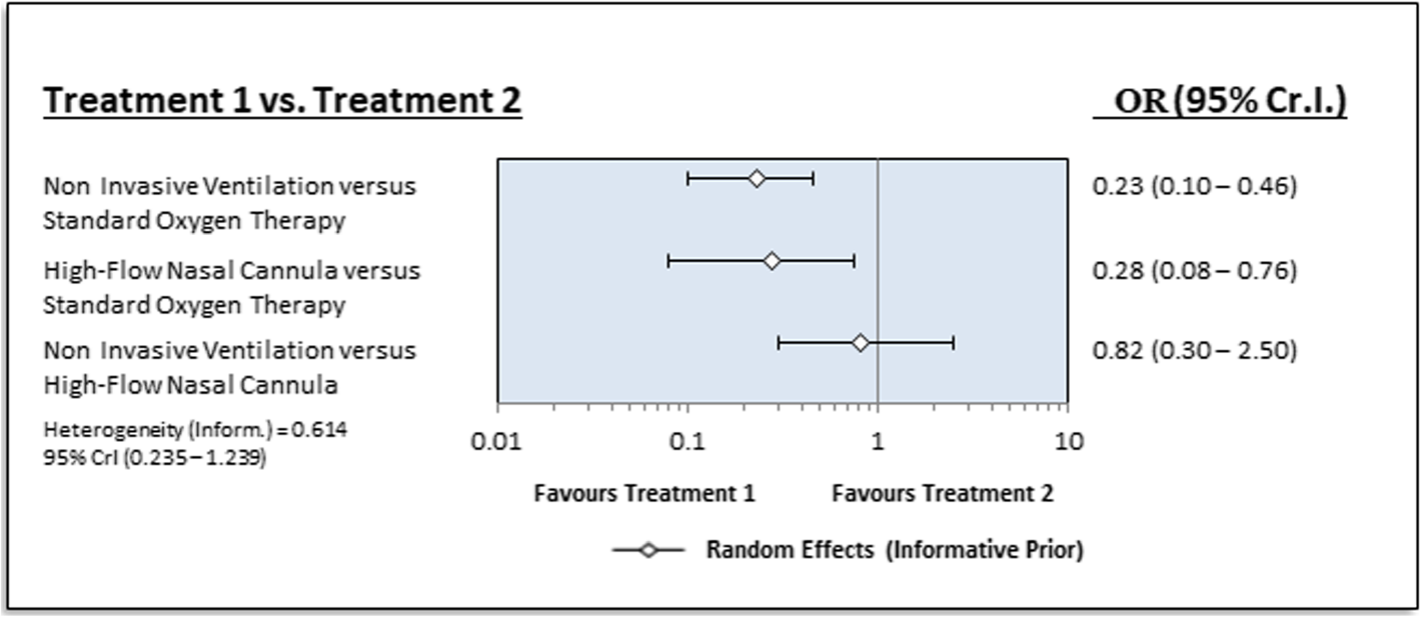
Forest plot for the rate of intubation between competing interventions

NIV and HFNC were associated with significant reductions in intubation rates when compared to standard oxygen therapy (OR 0.23; 95% Cr.I. 0.10–0.46) and (OR 0.28; 95% Cr.I. 0.08–0.76), respectively. However, there was no significant difference between HFNC and NIV with regard to the intubation rates (OR 0.82; 95% Cr.I. 0.30–2.50), Fig. 2.

**Fig. 3:**
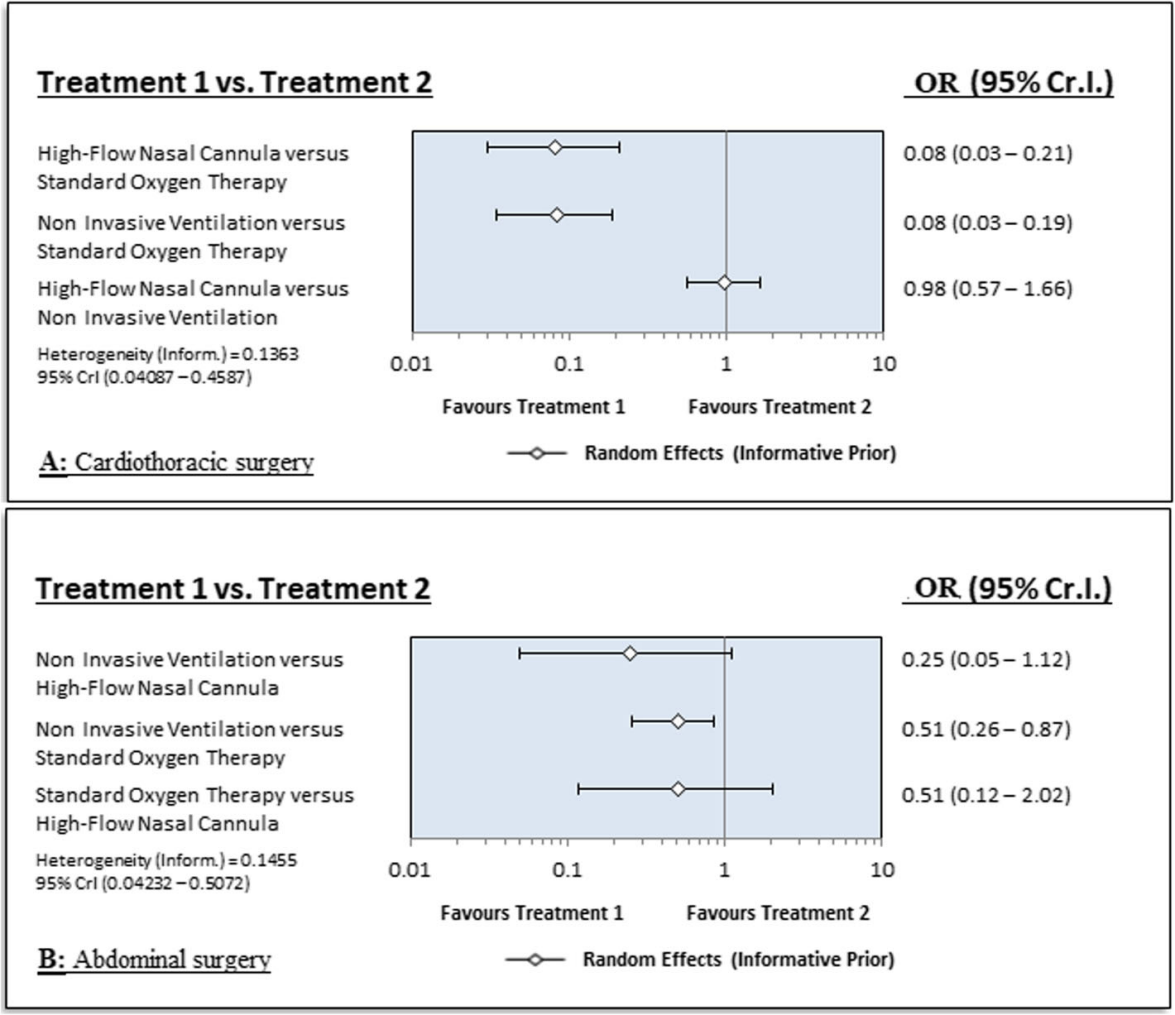
Forest plot for the rate of intubation between competing interventions following cardiothoracic surgery (**a**) and abdominal (**b**) surgery

Sensitivity analysis was performed by including only patients who developed acute hypoxemic respiratory failure (but not patients at increased risk), which showed similar results. In a subgroup analysis for patients undergoing cardiothoracic surgery, both NIV and HFNC were associated with a similar reduction in intubation rates compared with standard oxygen therapy (NIV vs standard oxygen (OR 0.08; 95% Cr.I. 0.03–0.19) and HFN vs standard oxygen (OR 0.08; 95% Cr.I. 0.03–0.21)) (Fig. 3a). However, in patients undergoing abdominal surgery, NIV (but not HFNC) was associated with significantly reduced intubation rates compared with standard therapy (NIV vs standard oxygen (OR 0.51; 95% Cr.I. 0.26–0.87)) (Fig. 3b).

In an exploratory meta-regression analysis, we found that higher PaCO2 was associated with lower risk for intubation when NIV was compared to standard oxygen therapy (*P* < 0.05) (Supplementary Figure 3).

#### Mortality

**Fig. 4:**
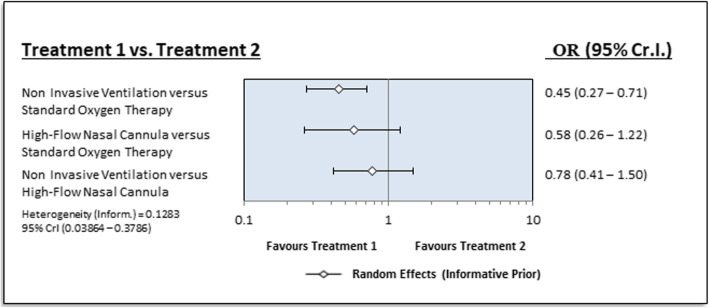
Forest plot for mortality between competing interventions

NIV was associated with a significant reduction of mortality in comparison with standard oxygen therapy (OR 0.45; 95% Cr.I. 0.27–0.71). Additionally, there was no significant difference between NIV and HFNC (OR 0.78; 95% Cr.I. 0.41–1.50) or HFNC and standard oxygen (OR 0.58; 95% Cr.I. 0.26–1.22) as shown in Fig. 4.

**Fig. 5:**
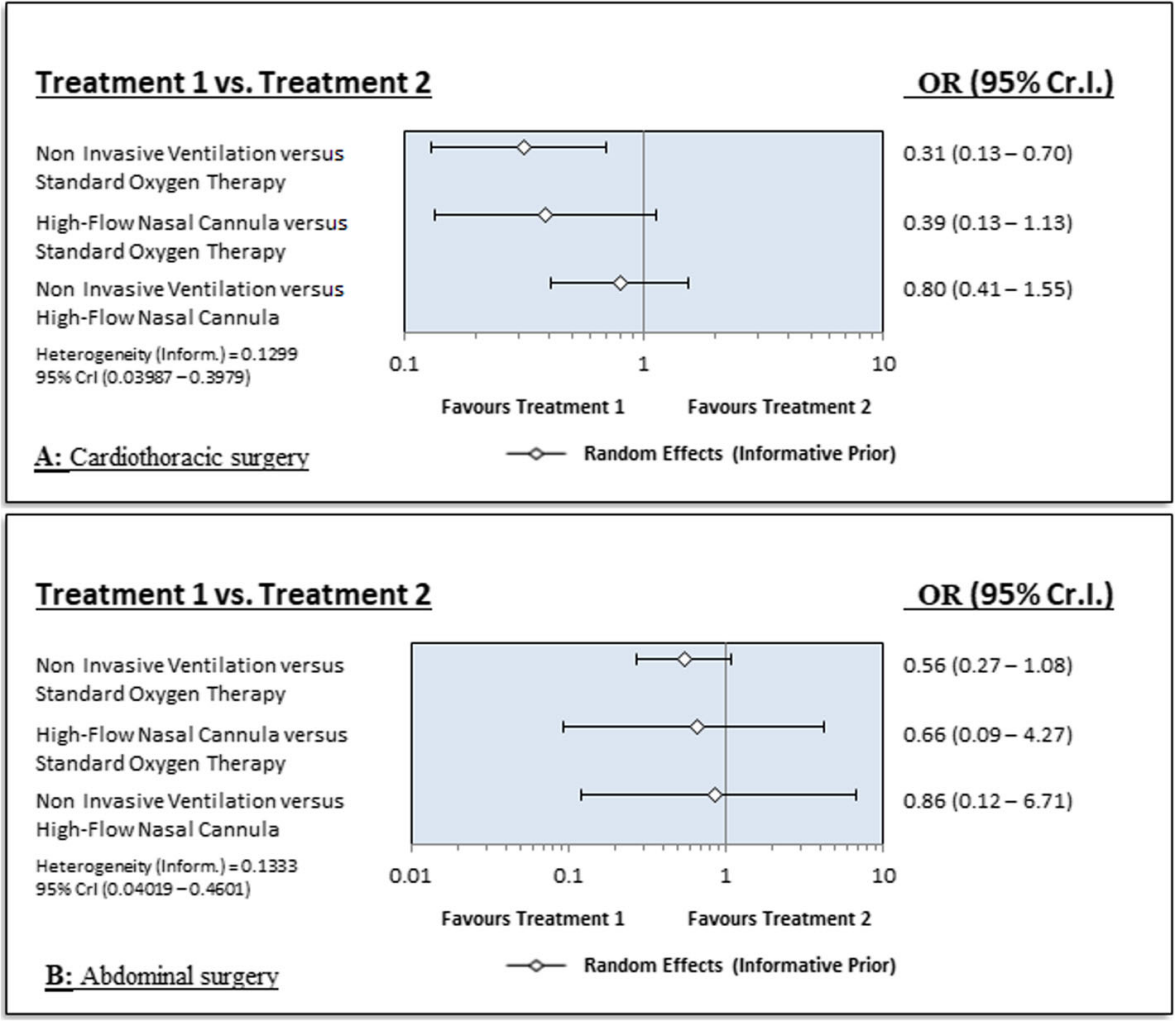
Forest plot for mortality between competing interventions following cardiothoracic surgery (**a**) and abdominal surgery (**b**)

In a subgroup analysis based on the type of surgery (cardiothoracic or abdominal), mortality benefit of NIV was limited to those undergoing cardiothoracic surgery compared with standard oxygen therapy (OR 0.31; 95% Cr.I. 0.13–0.70), unlike those undergoing abdominal surgeries (OR 0.56; 95% Cr.I. 0.27–1.08) (Fig. 5).

#### ICU-acquired infections

**Fig. 6:**
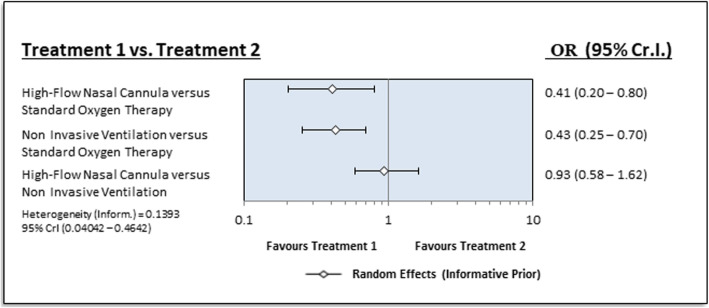
Forest plot for the incidence of ICU-acquired infections between competing interventions

HFNC and NIV were associated with a decreased risk for ICU-acquired infections in comparison with standard oxygen therapy (OR 0.41; 95% Cr.I. 0.20–0.80) and (OR 0.43; 95% Cr.I. 0.25–0.70), respectively. No significant difference was found between HFNC and NIV (Fig. 6).

#### Direct pairwise meta-analysis results

We have performed direct pairwise meta-analysis comparing NIV versus standard oxygen which showed consistent results of the network meta-analysis (Supplementary Figure 4). However, we did not perform the direct meta-analysis for HFNC vs NIV or HFNC vs standard oxygen because studies that compared directly between these interventions were one or two studies. Supplementary Figures 5 and 6 show the results of these individual studies for different outcomes.

## Discussion

In this first network meta-analysis comparing various oxygenation strategies in patients at risk for hypoxemic respiratory failure or established respiratory failure within 7 days of surgery, we have found that NIV and HFNC were associated with a significant reduction in intubation rates and ICU-acquired infections when compared to standard oxygen therapy. However, when compared to standard oxygen therapy, only NIV was found to have a mortality benefit in this patient population. We found HFNC and NIV to have no significant differences in the primary or secondary outcomes. Furthermore, in a subgroup analysis, patients undergoing cardiothoracic surgery had a significantly lower rate of intubation when treated with HFNC or NIV in comparison with standard oxygen therapy, but mortality was significantly lower in patients treated with NIV in comparison with standard oxygen therapy. Additionally, in patients with abdominal surgeries, only NIV was associated with a significant reduction in intubation rates compared to standard oxygen, but there was no significant difference in mortality between competing interventions.

Hypoxemia occurs frequently in the post-operative period and can lead to acute respiratory failure. Several factors play a role in the development of post-operative respiratory failure, including diaphragmatic dysfunction, retained secretions, and atelectasis and alveolar collapse which promote bacterial growth and infections [27–29]. Non-invasive ventilation (NIV) improves oxygenation by recruiting collapsed alveoli and increasing tidal volume participating in gas exchange without hemodynamic adverse events [1, 30]. However, previous studies and meta-analyses had not shown a significant reduction in intubation rates with prophylactic use of NIV after surgery, despite the reduction in the incidence of post-operative pulmonary complications [31–33].

Currently, NIV is recommended in the treatment of patients with post-operative respiratory failure according to the ERS/ATS guidelines [15]. Our results indicate that intubation rates and mortality are significantly lower in patient populations who are at an increased risk or have developed postoperative respiratory failure treated with NIV in comparison with standard oxygen. In our subgroup analysis, mortality benefit was only noted in patients undergoing cardiothoracic surgeries but not in abdominal surgeries. In an RCT examining NIV vs standard oxygen therapy in patients with respiratory failure after abdominal surgeries, Jaber and Antonelli found that NIV was associated with lower intubation rates, less days on mechanical ventilator, and significantly lower rates of healthcare-associated infections, including pneumonia. Although mortality rates were lower in the NIV group (14 vs 21%), the difference did not reach a statistical significance in their study [22]. Similarly, patients undergoing cardiothoracic surgery and treated with NIV for postoperative respiratory failure had lower rates of intubation and mortality when compared to patients treated with standard oxygen therapy [19, 24].

HFNC is a new oxygenation strategy that has been used more frequently in patients with respiratory failure. It is found to be more comfortable than NIV and can deliver concentrated oxygen reaching 100% with a high flow rate up to 60 mL/min [34, 35]. Furthermore, it can provide positive end-expiratory pressure up to 2–3mmHG [34, 35]. The use of HFNC has shown beneficial effects in patients who developed post-extubation respiratory failure or when used during intubation to prevent hypoxemia when compared to standard oxygen [36–38]. Additionally, Frat et al. found lower mortality rates with HFNC in comparison with NIV and conventional oxygen in patients with non-hypercapnic hypoxemic respiratory failure. However, other trials did not find differences between HFNC and standard oxygen therapy [39–42]. The use of HFNC in the post-operative period was investigated by several RCTs. In a large RCT involving more than 800 patients after cardiac surgery, the use of HFNC and NIV in the treatment of high-risk patients or those who had developed post-operative respiratory failure was similar between both interventions with similar intubation rates, mortality, and rates of hospital-acquired infections [20]. In our analysis, there was no difference between HFNC and NIV in intubation rates, mortality, and ICU-acquired infections. Similar results were also found in both subgroups (cardiothoracic surgeries and abdominal surgeries).

In addition, HFNC was associated with lower intubation rates in patients following cardiothoracic surgeries but not following abdominal surgeries when compared to standard oxygen therapy. This could be explained by the fact that in thoracic surgery, HFNC could minimize lung decruitment post-extubation by providing some level of continuous positive airway pressure through high-flow ventilation, though this positive pressure can be variable due to the leak around the nasal cannula and nonguaranteed closed mouth of the patients [26].

Furthermore, we found lower rates of infections with HFNC and NIV when compared to standard oxygen are attributed to lower intubation rates in both interventions, which avoids the need for mechanical ventilation and decreases catheter-associated infections.

Although there was no significant difference between HFNC and NIV with regard to rates of intubation, mortality, and ICU-associated infections, when each of these two strategies was compared to standard oxygen, NIV was associated with a survival benefit especially in patients who had cardiothoracic surgery. Additionally, there was a trend toward lower mortality in abdominal surgeries, but HFNC had no mortality benefit in the total patient population and both subgroups. Whether a lower number of patients included in the comparison between HFNC and standard oxygen or certain other factors could have contributed to the inability to detect a mortality benefit despite a significant reduction of intubation rates is needed to be addressed in further larger and well-controlled trials.

Nevertheless, due to the low events, further RCTs are needed to compare between both interventions in different types of surgeries to determine its effect on various long-term clinical outcomes and quality of life and also to examine whether certain patients’ risk factors could affect the beneficial effects of these interventions towards reduction of intubation rates and mortality.

## Limitations

Our analysis has several limitations. First, we were unable to perform analysis based on various risk factors, duration of surgery, severity scores, and different surgical types as we lack patients’ level data. Second, blinding of intervention and personnel was impossible given the nature of intervention. Third, there were few sample size and limited events, and therefore, larger trials and long-term outcomes are needed. Fourth, we used informative prior module for our analysis which could affect the results given the small number of included trials. Fifth, there was a significant time gap between included studies through which there was a significant development in the ICU management, preoperative and postoperative evaluation and care, supportive management, and criteria for admission to the ICU.

## Conclusion

Among patients who are at risk for developing post-operative respiratory failure, or have developed post-operative respiratory failure, the use of NIV was associated with reduced rates of intubation, mortality, and ICU-acquired infections in comparison with standard oxygen therapy. In addition, HFNC was associated with reduced rates of intubation and ICU-acquired infections but not mortality in comparison with standard oxygen. There was no significant difference between HFNC and NIV on the various studied clinical outcomes.

## Supplementary information

**Supplementary figure 1:.** Network geometry. Number of participants in each group represented with node size and the edge widths are proportional to the number of studies between different interventions. A = High-flow nasal cannula; B = Non-invasive ventilation; C = standard oxygen therapy.

**Supplementary figure 2:.** Risk of bias assessment based on authors’ judgment for each of the included RCTs. Blank items indicate unclear risk of bias.

**Supplementary figure 3: **Regression of PaCO2 on intubation rate between non-invasive ventilation and standard oxygen. Higher PaCO2 was associated with a lower risk for intubation with NIV use (*P <* 0.05).

**Supplementary figure 4:.** Direct meta-analysis results between NIV versus standard oxygen showing forest plots for intubation rate, mortality, and ICU acquired infections.

**Supplementary figure 5:.** Results of individual studies comparing between HFNC and standard oxygen for different outcomes.

**Supplementary figure 6:.** Results of individual studies comparing between NIV and HFNC for different outcomes.

## Data Availability

Data and materials are available and can be presented upon request.

## Abbreviations

NIV: Non-invasive ventilation
HFNC: High-flow nasal cannula
ICU: Intensive care unit
RCT: Randomized controlled trial
OR: Odds ratio
Cr.I.: Credible interval
