# Supplementary figures and images for: Effect of oxygenation modalities among patients with postoperative respiratory failure: a pairwise and network meta-analysis of randomized controlled trials
Source: J Intensive Care. 2020 Jul 17;8:51. doi: 10.1186/s40560-020-00468-x (PMC7366473; doi:10.1186/s40560-020-00468-x)

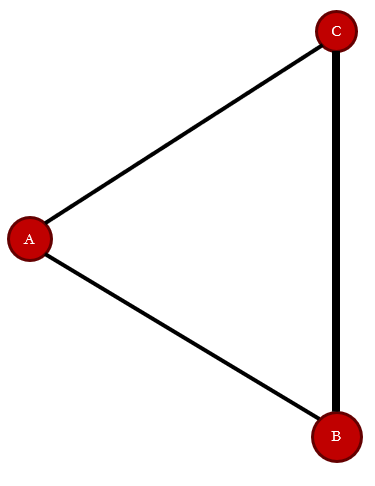

Supplement: Supplementary file 1 — Supplementary figure 1:. Network geometry. Number of participants in each group represented with node size and the edge widths are proportional to the number of studies between different interventions. A = High-flow nasal cannula; B = Non-invasive ventilation; C = standard oxygen therapy. [file 40560_2020_468_MOESM1_ESM.png]

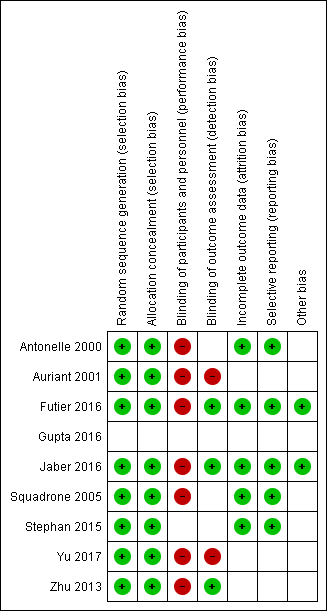

Supplement: Supplementary file 2 — Supplementary figure 2:. Risk of bias assessment based on authors’ judgment for each of the included RCTs. Blank items indicate unclear risk of bias. [file 40560_2020_468_MOESM2_ESM.png]

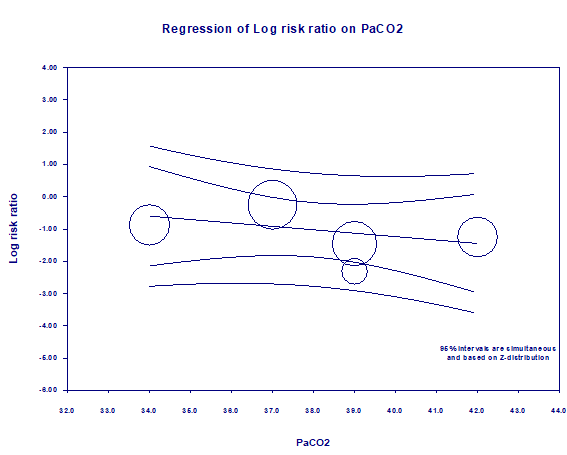

Supplement: Supplementary file 3 — Supplementary figure 3: Regression of PaCO2 on intubation rate between non-invasive ventilation and standard oxygen. Higher PaCO2 was associated with a lower risk for intubation with NIV use (P < 0.05). [file 40560_2020_468_MOESM3_ESM.png]

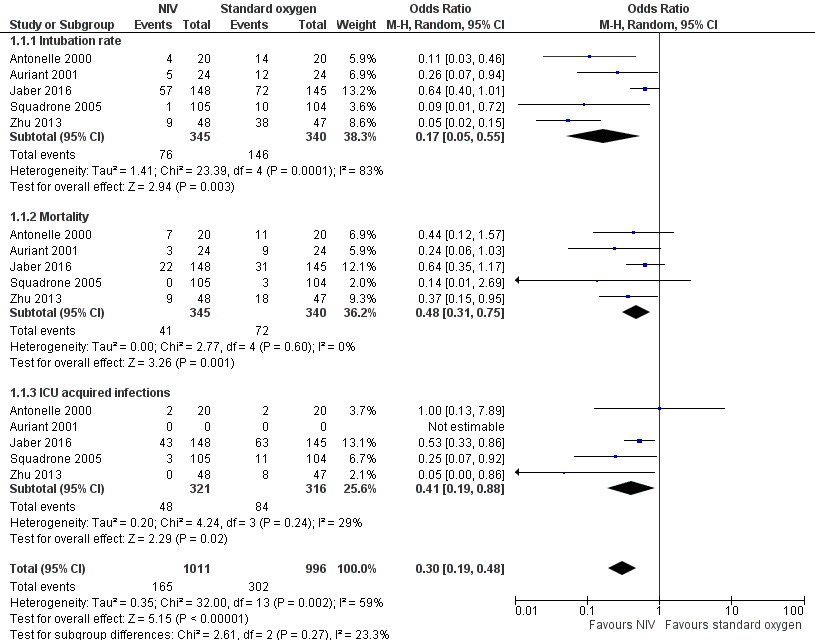

Supplement: Supplementary file 4 — Supplementary figure 4:. Direct meta-analysis results between NIV versus standard oxygen showing forest plots for intubation rate, mortality, and ICU acquired infections. [file 40560_2020_468_MOESM4_ESM.png]

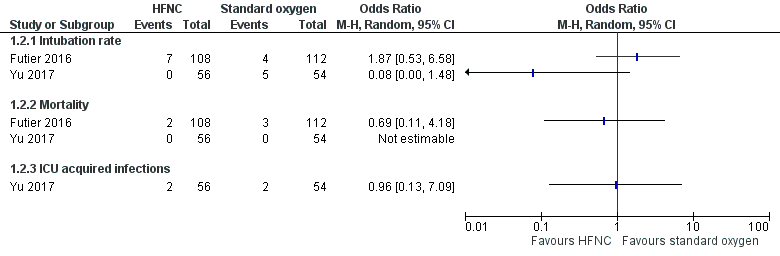

Supplement: Supplementary file 5 — Supplementary figure 5:. Results of individual studies comparing between HFNC and standard oxygen for different outcomes. [file 40560_2020_468_MOESM5_ESM.png]

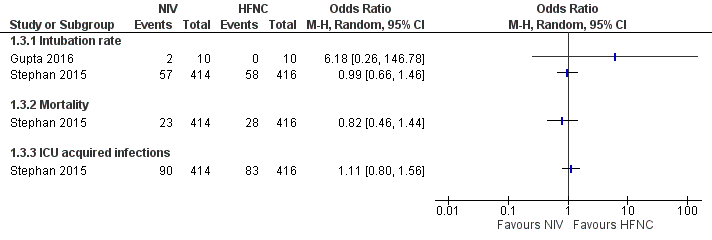

Supplement: Supplementary file 6 — Supplementary figure 6:. Results of individual studies comparing between NIV and HFNC for different outcomes. [file 40560_2020_468_MOESM6_ESM.png]
